# Liquid Chromatography–Charged Aerosol Detection for Characterization of Natural Toxin Reference Materials

**DOI:** 10.1021/acsmeasuresciau.6c00007

**Published:** 2026-03-16

**Authors:** Elliott J. Wright, Daniel G. Beach, Pearse McCarron

**Affiliations:** Metrology Research Centre, 6356 National Research Council of Canada, 1411 Oxford Street, Halifax, Nova Scotia B3H 3Z1, Canada

**Keywords:** reference materials, natural products, purity, stability monitoring, quantitation

## Abstract

Developing certified reference materials (CRMs) for natural toxins is challenging due to the scarcity of raw materials, their high toxicity, and structural complexity. Typically, purified toxins in microgram to low milligram amounts are quantitated as stock solutions using quantitative nuclear magnetic resonance spectroscopy (qNMR) and then accurately diluted to their final concentrations. With the limited sensitivity of qNMR, additional techniques are required for homogeneity assessment, quantitation, and stability monitoring of less concentrated solutions. Liquid chromatography with charged aerosol detection (LC–CAD) enables nonspecific detection of compounds down to nanogram-on-column levels. A reversed-phase LC–CAD method was developed that also incorporates MS and DAD. Large volume injection programs were implemented to improve detection for samples at low μg mL^–1^ concentrations. A retention index standard was used to monitor the overall method performance, while selectivity, repeatability, reproducibility, and response uniformity were evaluated using calibration solution CRMs from several toxin classes. Estimated repeatability was 3.9%, while the intermediate precision of normalized relative response factors was 1.6–8.5%. A relative response factor RSD of 13% was observed for 19 toxins from six different classes, but an average relative response factor of 4.8% within the class demonstrated that LC–CAD is highly suitable for within-class quantitation. The utility of LC–CAD is demonstrated at multiple stages of toxin calibration solution CRM development including purity assessment of stock materials, postcertification stability monitoring, and characterization measurements. The work highlights the important and broadly applicable role that LC–CAD can play in the development of vital reference materials for sample-limited natural products.

## Graphical abstract

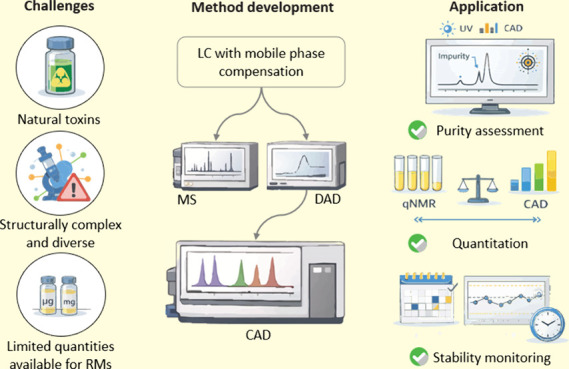

## 1 Introduction

Natural toxins produced by marine algae and freshwater cyanobacteria can be highly toxic and structurally complex, with some regulated at levels ranging from μg kg^–1^ to mg kg^–1^ in seafood and drinking water supplies.^1–3^ Reference materials (RMs) for these compounds and specifically certified reference materials (CRMs), are essential for monitoring programs to safeguard both consumer health and associated industries. Development of CRMs in accordance with international standards requires assessment of analyte purity, stability and homogeneity, while SI-traceable measurements are necessary to assign property values.^4–6^

One of the most significant challenges in CRM development for natural toxins is the limited availability of pure toxin materials. Sources may include purification from biomass collected during natural blooms or from laboratory culturing,^7,8^ isolation from contaminated shellfish^7,9^ and in limited cases, synthesis.^10^ Given the extensive effort required to obtain even milligram quantities of purified toxin, highly sensitive or nondestructive analytical techniques are essential. Quantitative nuclear magnetic resonance (qNMR) is a critical method in this regard, providing measurements in the low mg mL^–1^ range^8,11–13^ and SI traceability, even when standards of the compounds being analyzed are not available.^14^ For natural toxins, limited source material means that the final batch of CRM units is frequently prepared by quantitative dilution of a stock solution that was used for qNMR, because the technique lacks the sensitivity required to measure toxins at the final dilute CRM concentration.^12,13^ Mass spectrometry (MS) is one of the most commonly used methods for algal toxin analysis, however, calibrants of the toxin of interest are required since response between even closely related analytes in electrospray ionization can vary by up to 50%.^9^ A chemiluminescent nitrogen detection (CLND) method was developed, however its scope is limited to quantitating nitrogen-containing algal and cyanobacterial toxins.^15^

Universal detectors for liquid chromatography (LC) detect a diverse range of analytes^16^ and have been used in pharmaceutical^17^ and forensic applications,^18^ among others. Various universal detectors have been reported, each with its own technical nuances and limitations.^19^ Of these, a number of aerosol-based methods are available, including evaporative light scattering detection, condensation nucleation light scattering detection, and charged aerosol detection (CAD). Charged aerosol detection is of particular interest for natural toxins as it enables mass-sensitive detection of nonvolatile compounds and has been used for quantitation in the absence of available standards,^19^ for purity assessment,^20,21^ and for stability testing.^22^ The principles of CAD have been described in detail,^23^ along with best practices when using CAD quantitatively.^24^ Briefly, the LC eluent is nebulized to create aerosol particles, which are dried in an evaporation tube prior to unipolar diffusion charging through a corona discharge region, with the resulting charged particles measured using an electrometer. Charged aerosol detection response is impacted by mobile phase composition^23^ and analyte physicochemical properties.^25^ Therefore, method setup^26^ and careful consideration of the properties of analytes selected for calibration^27^ can have a significant bearing on the quantitative performance of CAD.

In this work, a reversed-phase LC–CAD method was developed as part of a multidetector configuration including diode array detection (DAD) and full scan mass spectrometry (MS). Performance characteristics of the method were assessed using a diverse suite of natural toxin calibration solution CRMs. Quantitative evaluation covered testing of calibration approaches, assessing relative response factors within and between toxin classes, and examining measurement repeatability and reproducibility. The method was used for toxin purity assessment, produced quantitative data in agreement with qNMR, and was successfully applied in postcertification stability monitoring over a number of years.

## 2 Experimental Section

### 2.1 Chemicals and Reagents

Mass spectrometry grades of formic acid were obtained from Honeywell (Oakville, ON, Canada) and ammonium formate was from Sigma-Aldrich (Oakville, ON, Canada). Deionized water was obtained from a Milli-Q Reference A+ water purification system (Millipore Corp., Billerica, MA, USA). Optima LC/MS grade acetonitrile was obtained from Fisher Scientific (Whitby, ON, Canada). Calibration solution CRMs for the algal and cyanobacterial toxins (Figure 1) were provided by the National Research Council (Halifax, NS, Canada) and included CRMs for AZA1, AZA2, AZA3, PTX2, [Dha^7^]­MC-LR, MC-LR, MC-RR, MC-LA, NOD-R, YTX, hYTX, OA, DTX1, DTX2, PnTX-G, 13desMeSPX-C and GYM. Stock solutions or candidate CRMs for AZA6, MC-LA, [d-Leu^1^]­MC-LY, MC-YR, DTX2 and YTX were also provided, along with a retention index standard (RM-RILC), used here for system performance monitoring. Details on the materials used are available on the NRC Digital Repository^28^ or in Table S1.

**Figure 1:**
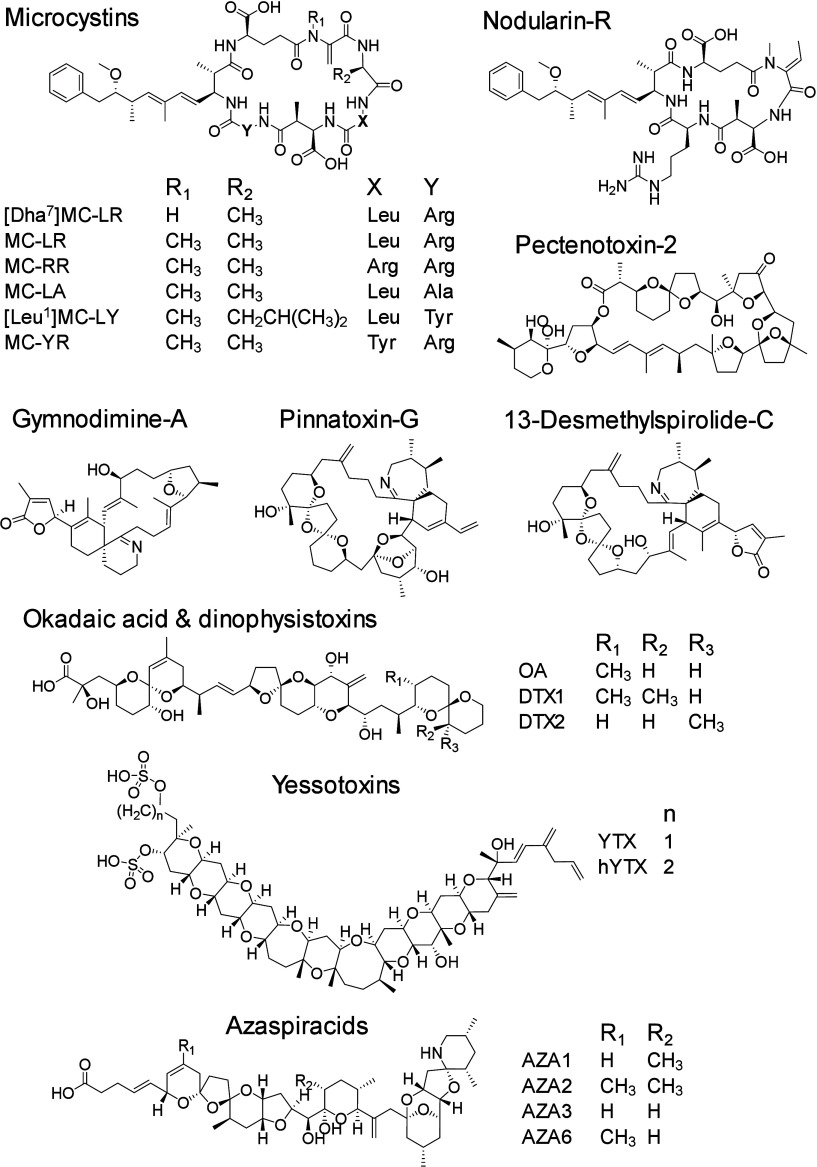
Chemical structures of algal and cyanobacterial toxins assessed in this study.

### 2.2 Instrument Configuration

An Agilent 1260 series HPLC system (Agilent Technologies, Palo Alto CA, USA) was used, consisting of two binary pumps (denoted as *analytical pump* and *supplementary pump*), an autosampler, column compartment, and a diode array detector (DAD). The HPLC system connected to a Thermo Corona VeoRS charged aerosol detector (Thermo Fisher Scientific, Waltham MA, USA) and a Thermo LTQ XL ion trap mass spectrometer (Thermo Fisher Scientific, Mississauga ON, Canada). The instruments were controlled using Xcalibur SII, with data processed in Tracefinder 4.1. A schematic of the LC and multidetector configuration is provided (Figure S1).

The mobile phase was water (A) or 95% acetonitrile (B), both containing 25 mM formic acid and 1 mM ammonium formate. A Thermo Hypersil BDS C_8_ column (3 μm, 150 × 2.1 mm) was maintained at 25 °C. The analytical pump ran a linear gradient at 0.200 mL min^–1^ from 20–100% B over 20 min, holding for 12 min, before re-equilibration. Gradient compensation was carried out by teeing in an inverse gradient from the supplementary pump to the column eluate. The supplementary pump ran at 0.200 mL min^–1^, holding initially at 100% B for 4.8 min and then running a linear gradient to 20% B over 20 min, holding for 7.2 min, before re-equilibration. The 4.8 min hold on the supplementary pump gradient was determined experimentally by adding 0.05% (v/v) acetone to mobile phase A, and adjusting gradient start times to achieve a flat UV baseline. A six-port switching valve delivered flow directly from the autosampler to the column during analysis and switched at 32 min (position 1 → 6) to direct flow through two Phenomenex SecurityGuard C_18_ cartridges (4 × 3.0 mm) located prior to the analytical column, switching back to the sample analysis position (1 → 2) at 39.9 min. The combined pump flow was split at approximately 1:9 (measured volumetrically) by restricting through 63.5 μm ID tubing for the MS, with the remainder of the flow directed through the DAD to CAD using 127 μm ID tubing. Flow was diverted to waste between 0–5 and 32–40 min for CAD and MS detectors using their integrated switching valves. Different injection programs were used depending on CRM concentration (Tables S1, S2) ranging from 10–72 μL, with 1 μL injections for RM-RILC. CAD settings; 2 s filter, power function set to 1.0, data rate 10 Hz and evaporation temperature set to 25 °C. DAD settings; UV absorbance monitored at 265 nm wavelength (8 nm bandwidth) with a reference wavelength of 435 nm (80 nm bandwidth), data collected at 2.5 Hz, and every second spectrum stored from 190–400 nm (2 nm steps). The MS used a heated electrospray ionization source with a 34-gauge spray needle, a capillary temperature of 240 °C, a source heater temp of 70 °C and sheath and auxiliary gas flows of 30 and 5 (arbitrary units), respectively. Detection in full-scan mode used polarity switching from *m*/*z* 200–1200, and automatic gain control target of 30,000 ions. Additional instrument settings are given in Table S3.

### 2.3 Performance Evaluation and Application

Limits of detection (LOD) and limits of quantitation (LOQ) were estimated using signal-to-noise ratios of 3 and 10, respectively. To assess calibration models, CRM-MCRR was injected from 0.5 to 10 μL and the CAD power function was increased from the 1.0 default setting to 1.1, and 1.2. RM-RILC was used as a quality control sample and injected periodically throughout each sequence (≥9 injections) to monitor performance of gradient compensation and assess repeatability. Intermediate precision was tested by choosing an injection volume for each toxin calibration solution to deliver 24–155 ng on a column over 2–5 independent experiments. Relative response factor (RRF) was calculated by dividing the peak area of each analyte by the injected nanogram mass on column, and normalized to the RRF of okadaic acid (OA) to correct for day-to-day variability in absolute instrument response (nRRF_*x*_, with x representing the analyte of interest). Within-class quantitation was assessed by measuring calibration solution CRMs of structural analogues (≥3 injections for each) using injector programs (Table S1, S2) to closely match analyte masses on column. Uncertainties were calculated as a sum of squared errors with contributions from precision of calibrant and analyte measurements as relative standard deviations (RSDs), and the relative standard uncertainty from the calibrant CRM.

Stock solutions for CRMs were measured using qNMR,^29^ with information for individual CRMs available in the NRC Digital Repository.^28^ Uncertainty contributions were included for calibrant preparation, measurement precision of both analyte and calibrant integrals, and gravimetric dilution of the qNMR stock to the final ampule concentration.

## 3 Results and Discussion

### 3.1 Method Development

When the instrument configuration was established, the fundamental properties of each detector were considered. The CAD and MS are both sample destructive detectors, with mass-dependent and concentration-dependent response, respectively. Flow splitting (9:1) directed the majority of the column eluate to the CAD. The supplementary pump flow-path had a lower dead volume, so a 4.8 min delay was used to correct for the difference in dwell volume. The supplementary flow maintained a consistent mobile phase composition to the detectors, and also served to improve sensitivity by maximizing the proportion of organic modifier in the resulting flow to the CAD detector, enhancing the mass transfer of analyte during nebulization.^23^

Minimizing and stabilizing the CAD background current is critical for consistent signal-to-noise ratios; therefore, reagent purities and buffer concentrations require careful consideration. The mobile phase was a modification of a formic acid/ammonium formate buffer commonly used for lipophilic algal toxins at half the typical concentration,^30^ and resulted in a CAD background of approximately 1–2 pA. Further reduction in buffer concentration negatively impacted chromatographic performance. Impurities observed in chromatograms with no sample injected were found to be mobile phase reagent impurities and system contaminants concentrating on the column during analysis and run re-equilibration. A number of approaches were considered to reduce the intensity of these peaks. Utilizing a switching valve, C_18_ guard cartridges were placed in the flow path prior to the LC column (see Figure S1) in an effort to capture mobile phase contaminants during re-equilibration prior to the next injection. However, low levels were still detected (blank trace in Figure 2) due to the concentration on-column and subsequent elution. The switching valve was engaged at 32 min to provide 100% B at an elevated flow rate to wash off contaminants captured during the previous injection’s re-equilibration. Ultimately, best practices in mobile phase preparation including use of fresh reagents and well rinsed glassware, along with reducing method re-equilibration time, were the most effective in terms of minimizing baseline impurities. Given the complex instrument configuration consisting of two pumps and three detectors, a quality control sample was used to assess system suitability and, where appropriate, evaluate specific method performance criteria. RM-RILC^31^ is a retention index standard containing 20 *N*-alkylpyridinium-3-sulfonates (NAPS) of different alkyl chain lengths that elute as a series of peaks across the chromatography gradient (Figure 2). The individual NAPS peaks are observed in both positive and negative polarity MS, contain a UV chromophore (λ_max_ = 265 nm), and are present at concentrations easily detectable by CAD.

**Figure 2:**
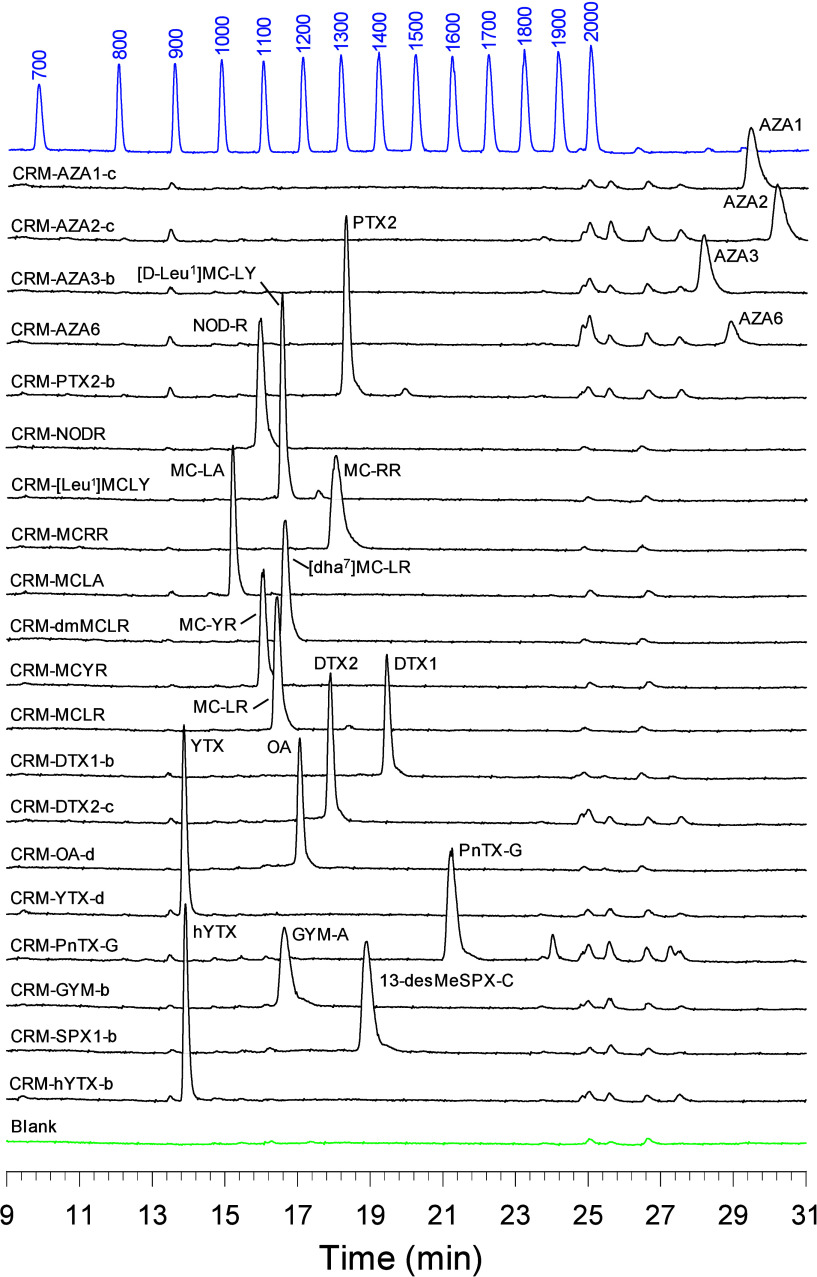
LC–CAD chromatograms of representative toxin CRMs (black) normalized to YTX (14.5 pA), RM-RILC (blue) showing NAPS peaks labeled with their assigned retention index values (700–2000), and a 50 μL injection of methanol as a blank (green). CRM injection programs (Table S1 and S2) delivered 35–123 ng on column.

Large volume injection programs were developed (Table S2) to ensure the mass of analyte on column exceeded the method LOQ. Injector program accuracy was confirmed by comparing 5 μL standard injections of RM-RILC to 25 and 50 μL injections of 5-fold and 10-fold methanol dilutions, respectively, demonstrating equivalent results (Figure S2).

### 3.2 Performance Characteristics

The column and mobile phase selections, along with the gradient used, provided good separation of the different toxin classes (Figure 2). While the measures described above minimized baseline noise and removed some background interference, trace level impurities from the CRMs themselves also present a risk of interference. A number of impurities eluted in close proximity to the AZAs (Figure 2), so a lower injection volume was used to reduce relative peak sizes and improve resolution. Using representative analytes from each toxin class, the method LOD was estimated at ≤ 2.0 ng on column.

A variety of calibration models have been reported for LC–CAD including linear, quadratic,^32^ and log–log linear models.^33^ Using MC-RR, which had a linear response by UV with no detectable bias in the residuals (Figure 3A), a variety of calibration models were compared. Up to ± 5% bias was observed in all CAD calibration models tested, at levels above the LOQ (Figure 3 and Figure S3). All CAD calibration models tested showed systematic trends in residual plots, demonstrating the need for careful selection of the calibration approach for high-accuracy quantitation. For this application, single-point calibration using a standard of matched signal intensity was chosen to minimize bias. The detector’s power function value (PFV) setting has been used to linearize response,^34^ but in this work, increasing the PFV value reduced sensitivity (Figure 3B), and was not considered further.

**Figure 3:**
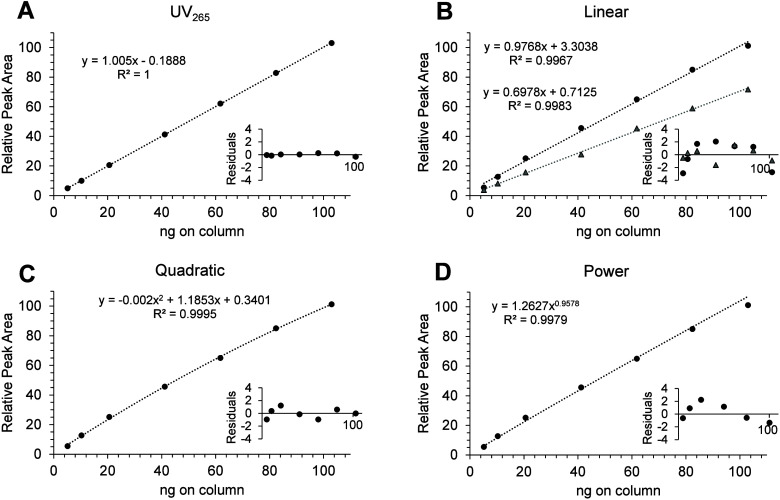
Calibration models tested using MC-RR response with linear regression for UV detection (A), and linear regression for CAD (B) with power function value (PFV) set to either 1.0 (black circles) or 1.1 (gray triangles). CAD using default PFV = 1 with quadratic regression (C) and power function (D). Plot insets show residuals, with all peak areas normalized to the maximum intensity in pane B

Experiment repeatability was estimated for each analyte, and the mean of their RSDs was comparable to the average RSD (*n* ≤ 14) for the area of NAPS peaks RI 700–2000 in RM-RILC, which ranged from 2.8 to 6.4% (mean 3.9%) over five separate runs. This precision was comparable to previously reported LC–CAD^35,36^ and LC–MS/MS methods^30,37,38^ and demonstrates LC–CAD’s suitability for precision-based studies (e.g., stability and homogeneity analysis) at the low μg mL^–1^ concentrations typical for sample-limited natural product RMs.

To evaluate intermediate precision, injection volumes were selected to deliver approximately 100 ng on the column for each analyte. As the majority of the tested toxin CRMs were prepared in methanol, the injector programs helped maintain the chromatographic peak shape by increasing the aqueous composition of the injected solution. Up to five independent experiments were conducted for all analytes and used to determine response factors relative to OA (nRRF_*x*_) (Figure 4). The nRRF RSDs varied from 1.6 to 8.5% (mean 4.6%) for individual analytes and from 5.0 to 11% within class, while an RSD of 13% for nRRFs across all toxins indicated a difference in response between analyte classes (Figure 4). This variability is consistent with observations by Liu et al.^39^ who showed 17% overall variability between CRMs of nonvolatile compounds, while within-class variability for saccharides and bisphenols was in the range of 2 to 8%. Stanaszek et al.^18^ showed variability of ± 30% for a range of fentanyl derivatives, with decreased variability if analogues bearing specific functional groups were omitted.

**Figure 4:**
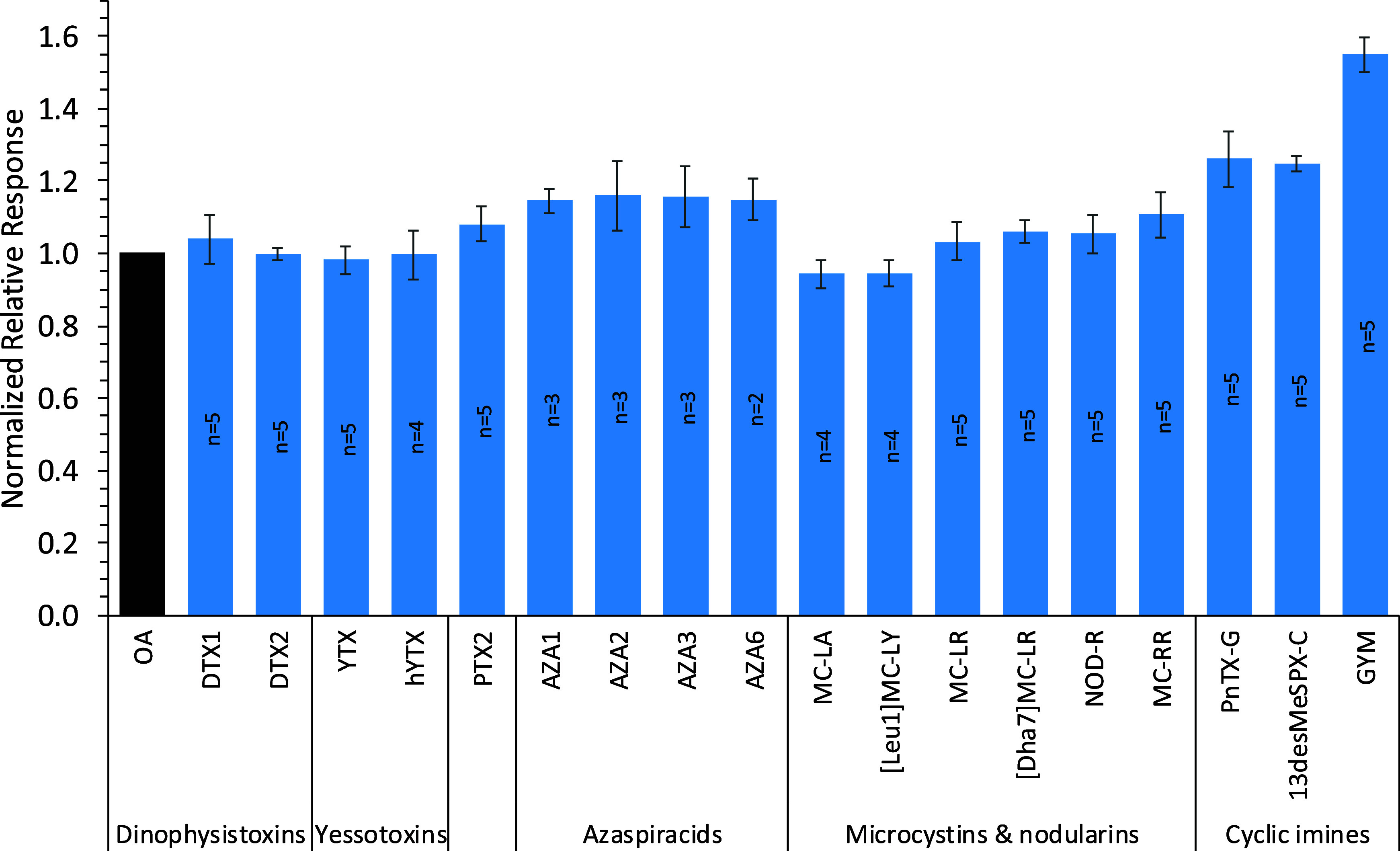
Relative response factors of algal and cyanobacterial toxins normalized to the response of OA (Table S1). Data acquired over series of independent experiments (*n* = 2–5). Error bars represent ± one standard deviation about the nRRF mean.

Yessotoxins, dinophysistoxins, pectenotoxins, and azaspiracids (Figure 1) are all polyether derivatives, with only minor within-class structural modifications for the analytes studied here, such as the placement and presence of methyl groups. The response factor agreement within these classes (5.0–5.7% RSD) was within the range observed for the individual toxins, indicating that these structural modifications do not elicit a detectable change in CAD response. The higher variability for microcystins and nodularin (7.1% RSD) is explained by the presence of varying numbers of arginine residues (0, 1 or 2) in some structures (Figure 1), which would bear a positive charge at the mobile phase pH used, thereby enabling pairing with formate ions in the mobile phase and resulting in a higher observed response. However, splitting microcystins into subclasses bearing the same number of arginine residues improved agreement considerably (e.g., MC-LA and [Leu^1^]­MC-LY with 3.7% RSD, or MC-LR, [Dha^7^]­MC-LR and NOD-R with 4.9% RSD). Like the AZAs, the cyclic imines contain a positive charge under the analysis conditions used, partially explaining their increased nRRFs relative to neutral compounds. The cyclic imines showed the highest within-class variability (11% RSD); however, it is noted that these toxins are generally classified based on shared possession of a cyclic imine, despite other significant structural differences. Spirolides and pinnatoxins bear the most similarities (Figure 1), while gymnodimine is markedly different in terms of size and atom connectivity. Omitting gymnodimine, there was a good nRRF agreement between the other cyclic imines (4.2% RSD).

### 3.3 Purity

For natural products where source material is scarce, purity assessment is typically conducted using a combination of methods with a focus on characterizing detectable impurities and determining whether those impurities interfere during RM value assignment or use.^12,15^ This is fundamentally different to the approach typically taken for pure substance CRMs, where impurities are quantitated and the purity of the substance is certified^40^ rather than its concentration in solution. The method developed here was applied to investigate impurities in a stock solution for a candidate MC-LA CRM. A 10-fold dilution of the stock was injected at 0.1 μL to bring the MC-LA peak areas down to the level of the detected impurities in injections of the neat MC-LA CRM stock. This served to minimize calibration bias, leaving differences in RRFs between the compounds as the largest remaining uncertainty.^21^

The multidetector configuration combining CAD, DAD and MS provided data that helped in determining whether observed impurities were structurally related. Two impurity peaks were detected at 16 min (1) and 17 min (2), with peak areas <0.5% relative to MC-LA (Figure 5). Full scan MS data (Figure S4) revealed that peak 1 was two coeluting impurities of approximately equal intensity, which gave adduct distributions similar to that of MC-LA. An *m*/*z* difference from MC-LA suggested one was a dehydrated MC-LA, and that the other was a methyl ester possibly formed during storage in 50% methanol. Peak 2 did not show an adduct distribution consistent with that of MC-LA, and also gave a different UV absorption profile (λ_max_ = 260 nm vs 244 nm for MC-LA), suggesting it was less structurally similar to MC-LA. While the impurities detected in this example were at trace levels, it illustrates how CAD with complementary data from other detectors is useful for purity assessment, providing an approach to accurately correct data from techniques such as qNMR in scenarios where impurities interfere with integrals being used for quantitation.

**Figure 5:**
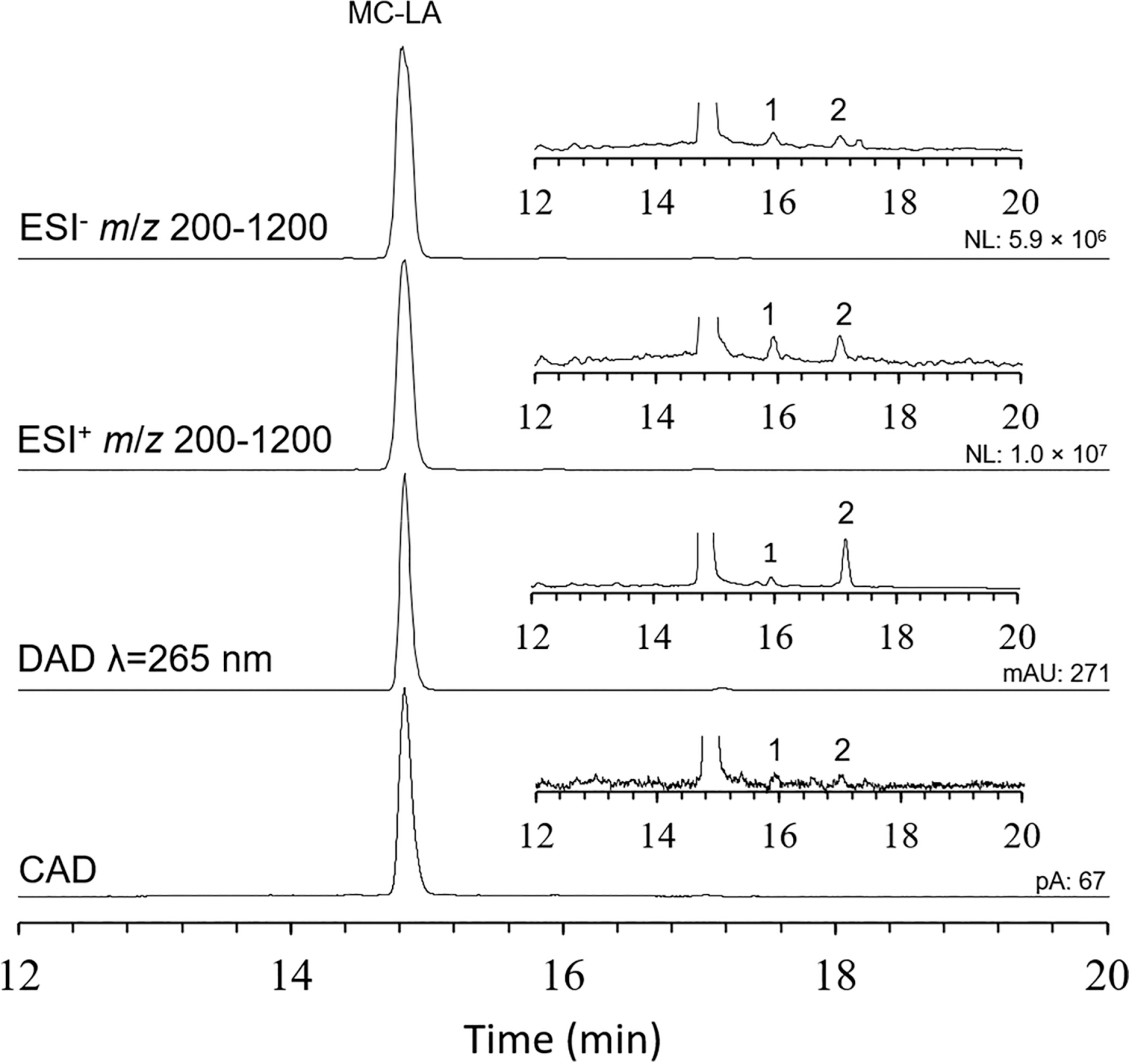
Purity analysis of MC-LA CRM stock solution by LC–MS–DAD–CAD. Inset chromatograms at 60× zoom on vertical axis show the minor impurities (1 and 2) detected.

### 3.4 Stability Monitoring

Stability monitoring of CRMs during long-term storage^4^ can present a significant challenge for natural product CRMs. Often, final CRM units are too dilute for analysis by qNMR and CRMs are not available from external providers for calibration of more sensitive techniques. LC–CAD is an attractive approach for long-term stability monitoring in these scenarios. The basic evaluation of a stability monitoring experiment according to international standards^6^ is

1 $${|x}_{CRM}-x_{mon}|\leq k\sqrt{\left(u_{CRM}^{2}+u_{mon}^{2}\right)}$$

where *x*_*CRM*_ and *x*_*mon*_ are the certified concentration and experimentally determined concentration from monitoring, respectively, *k* is a 95% confidence interval coverage factor, and *u*_CRM_ and *u*_mon_ the standard uncertainties of the CRM and monitoring method, respectively. The reproducibility of nRRFs was shown to be consistent between runs (mean nRRF_*x*_ RSD = 4.7%), and can be monitored for consistency over the CRM lifetime using an adaptation of eq 1:

2 $$n{RRF}_{x}-{nRRF}_{xmon}\leq k\sqrt{\left(u_{nRRFx}^{2}+u_{nRRFxmon}^{2}\right)}$$

where nRRF_*x*_ is the experimentally determined mean normalized relative response for analyte *x* from performance evaluation studies. Its uncertainty, *u* ^2^_nRRFx_, the variance about the nRRF_*x*_ results from the reproducibility trials, while nRRF_x mon_ is an OA-normalized monitoring measurement with an uncertainty *u* ^2^_nRRFx mon_ representing the variance about nRRF_x mon_ determination. The values of both variance terms in eq 2 are obtained by combining precision estimates (i.e., relative RSDs) of both OA and analyte *x* as a sum of squares.

A more robust estimator for these variance terms can be obtained using the mean repeatability estimate from RM-RILC (RSD_r_), which has 14 peaks eluting across the retention time range (Figure 2) and was injected numerous times throughout each sequence. For example, when this uncertainty estimate is used to represent precision for both OA and the analyte measurements for a monitoring point, the variance term can be further simplified to

3 $$u_{nRRFxmon}^{2}\approx \sqrt{2u_{RSDr}^{2}}$$

Example control charts for select CRMs over more than an 8-year period are shown in Figure 6.

**Figure 6:**
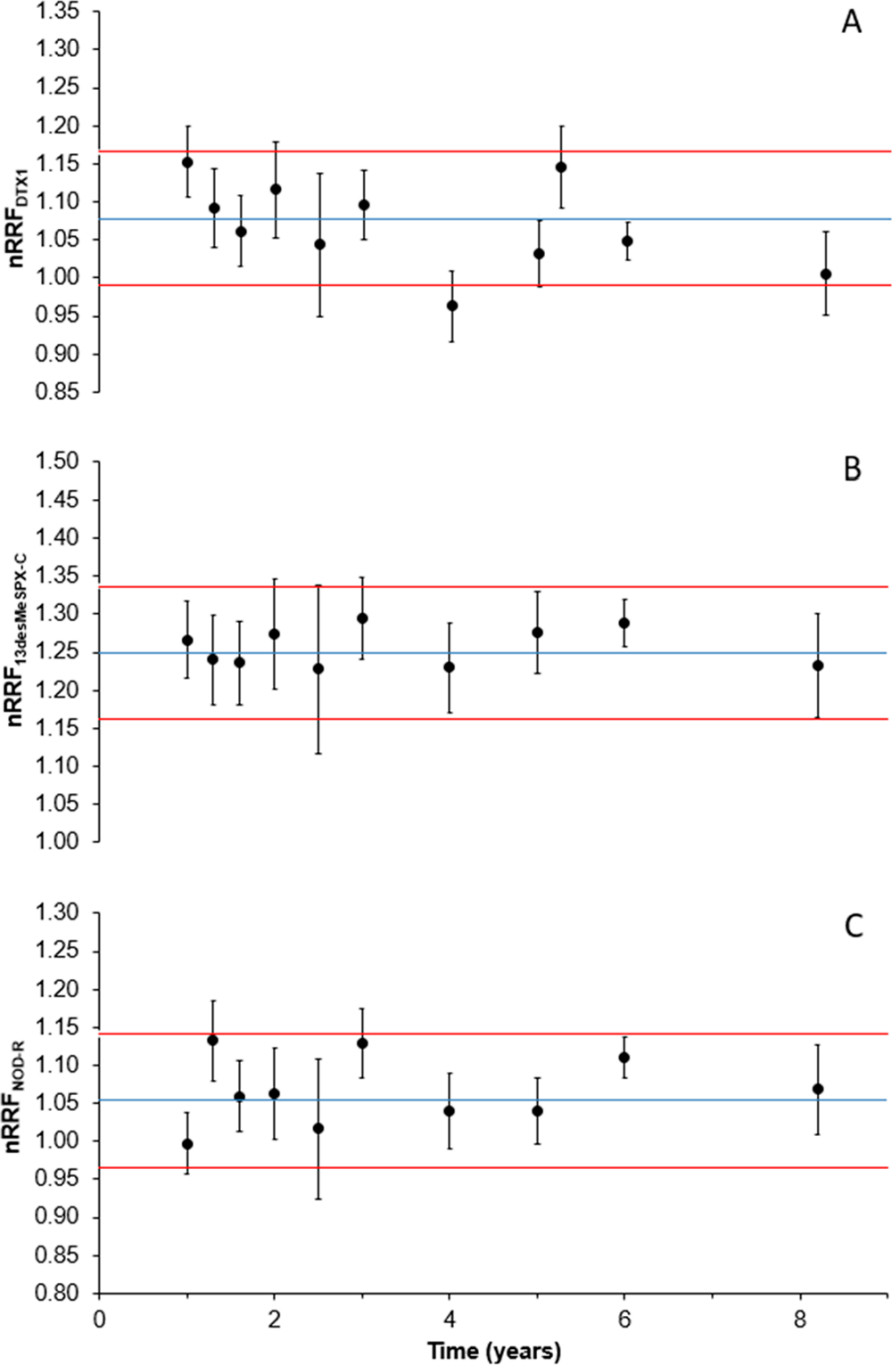
Control charts for continued stability monitoring of CRMs of dinophysistoxin-1 (A), 13-desmethylspirolide-C (B) and nodularin-R (C). The blue line represents the experimentally determined mean nRRF_*x*_ (*n* = 5 analyses), red lines are the control limits (eq 2), with error bars on monitoring points calculated using eq 3.

### 3.5 Quantitation

LC–CAD was applied in the characterization of calibration solution CRMs using closely related toxin analogue CRMs as calibrants. Certification measurements were performed by ^1^H qNMR with external calibration using benzoic acid or caffeine on stock solutions prior to dilution to final ampule-level concentrations. LC–CAD provided measurements at final CRM unit concentrations, which were particularly useful in the case of new materials when no previous CRM lot was available for calibration by LC–MS (e.g., AZA6, [Leu^1^]­MC-LY, and MC-YR). Table 1 shows excellent agreement between results from CAD and qNMR experiments on candidate CRMs for five algal and cyanobacterial toxins. These results are promising for a broader use of LC–CAD when carefully selected structurally similar calibrants are used with single-point matched mass-on-column injections.

**Table 1:** Comparison of Results from qNMR and Quantitative LC–CAD Measurements on Candidate Algal and Cyanobacterial Toxins CRMs

		Result ± standard uncertainty (μmol/kg)	
Candidate CRM	CRM for CAD calibration	CAD	qNMR
YTX-d	hYTX-b	5.3 ± 0.1	5.2 ± 0.1
DTX2-c	DTX1-c	6.0 ± 0.4	6.0 ± 0.1
AZA6	AZA1-c	0.71 ± 0.02	0.74 ± 0.04
[Leu^1^]MC-LY	MC-LA	8.0 ± 0.4	8.1 ± 0.1
MC-YR	MC-LR	4.4 ± 0.4	4.5 ± 0.1

## 4 Conclusions

Here, the utility of LC–CAD has been demonstrated in multiple stages of calibration solution CRM development and characterization, with a particular focus on natural toxins for which source materials are limited. The LC–CAD method was developed and evaluated for repeatability, reproducibility, calibration models, detection limits, and response factors. Method precision is suitable for homogeneity and stability studies, while the reproducibility of nRRFs demonstrated that LC–CAD is robust for postcertification monitoring of algal and cyanobacterial toxin CRMs. The work highlighted the importance of assessing calibration models used for CAD in order to minimize calibration bias with matched-level single point calibration being an effective strategy. The multidetector configuration, incorporating DAD and MS alongside CAD, provides further advantages for purity assessment of candidate CRMs. Finally, the method was applied in the characterization of new calibration solution CRMs from five different toxin classes, and strong agreement was observed with qNMR results. The approach is generally applicable in the development and characterization of sample limited natural product calibration solution RMs.

## Supplementary Material



## Notes

Information on CRMs used, liquid chromatography injector programs, instrument parameters and configuration, and supporting figures and data (PDF)
