# Supplementary material for: Liquid Chromatography–Charged Aerosol Detection for Characterization of Natural Toxin Reference Materials
Source: ACS Meas Sci Au. 2026 Mar 16;6(2):535–43. doi: 10.1021/acsmeasuresciau.6c00007 (PMC13087948; doi:10.1021/acsmeasuresciau.6c00007)
Supplement: Supplementary file 1 [file tg6c00007_si_001.pdf]

Supporting Information

Liquid Chromatography–Charged Aerosol Detection for Characterization of Natural Toxin Reference Materials

Elliott J. Wright, Daniel Beach, Pearse McCarron*

Metrology Research Centre, National Research Council of Canada, 1411 Oxford Street, Halifax, Nova Scotia, B3H 3Z1, Canada

* Corresponding author:

Tel +1 902-426-6182;

Fax +1 902-426-5426;

Email: pearse.mccarron@nrc-cnrc.gc.ca

Contents

Table S1. Natural toxin reference materials assessed in this study………………………………...3

Table S2. Large volume injection programs ……………………………………………………..4

Table S3. LTQ XL electrospray ionization (ESI) tune parameters ………………………………4

Figure S1. Schematic of LC-MS-DAD-CAD instrument configuration used in this study ...……5

Figure S2. Normalized UV results from testing of RM-RILC dilutions ………………………..6

Figure S3. % Difference between measured and expected ng of MC-RR compared with linear UV data from DAD ……………………………………………………..…………………7

Figure S4. Analysis of MC-LA stock by full scan MS (LTQ XL) ..……………………………...8

Table S1. Natural toxin calibration solution CRMs assessed in this study. For instances where multiple versions of CRM were used for the same compound an additional row is added to provide the CRM name, property value and uncertainty. Additional material details are available on the NRC Digital Repository (https://nrc-digital-repository.canada.ca/eng/home/).

Compound	CRM	CRM Diluent	$x_{CRM} \pm U_{CRM}$ [µg mL^{-1}][a]	Formula	Molecular Weight (Da)	Injection Volume (µL)[d]	Injected ng on-column
Azaspiracid-1	CRM-AZA1-b	CH_3OH	1.30 ± 0.07	$C_{47}H_{71}NO_{12}$	842.1	72	94
	CRM-AZA1-c	CH_3OH	1.22 ± 0.06			50[c], 25[f]	61, 31[f]
Azaspiracid-2	CRM-AZA2-b	CH_3OH	1.22 ± 0.06	$C_{48}H_{73}NO_{12}$	856.1	72	88
	CRM-AZA2-c	CH_3OH	1.19 ± 0.06			50[c]	60
Azaspiracid-3	CRM-AZA3-b	CH_3OH	1.18 ± 0.05	$C_{46}H_{69}NO_{12}$	828.1	72, 50[c]	85, 59
Azaspiracd-6	CRM-AZA6	CH_3OH	0.48 ± 0.04	$C_{47}H_{71}NO_{12}$	842.1	72[f], 50[c]	35[f], 24
Pectenotoxin-2	CRM-PTX2-b	CH_3OH	4.40 ± 0.13	$C_{47}H_{70}O_{14}$	859.1	25	110
[Dha[7]]Microcystin-LR	CRM-dmMCLR	1:1 CH_3OH:H_2O	9.40 ± 0.44	$C_{48}H_{72}N_{10}O_{12}$	981.2	10	94
Microcystin-LR	CRM-MCLR	1:1 CH_3OH:H_2O	10.1 ± 0.4	$C_{49}H_{74}N_{10}O_{12}$	995.2	10	101
Microcystin-RR	CRM-MCRR	1:1 CH_3OH:H_2O	10.3 ± 0.5	$C_{49}H_{75}N_{13}O_{12}$	1038.2	10	103
Microcystin-LA	CRM-MCLA	1:1 CH_3OH:H_2O	4.69 ± 0.19	$C_{46}H_{67}N_7O_{12}$	910.06	20	94
[Leu[1]]Microcystin-LY	CRM-[Leu[1]]MCLY	1:1 CH_3OH:H_2O	7.77 ± 0.30	$C_{55}H_{76}N_7O_{13}$	1043.2	20, 15	155, 117
Microcystin-YR	CRM-MCYR[e]	1:1 CH_3OH:H_2O	N/A[e]	$C_{52}H_{72}N_{10}O_{13}$	1045.2	20	
Nodularin-R	CRM-NODR	1:1 CH_3OH:H_2O	10.3 ± 0.4	$C_{41}H_{60}N_8O_{10}$	825.0	10	103
Yessotoxin	CRM-YTX-c	CH_3OH	4.9 ± 0.2	$C_{55}H_{82}O_{21}S_2$	1143.4	25	123
	CRM-YTX-d	CH_3OH	4.74 ± 0.26			25	119
Homo-Yessotoxin	CRM-hYTX-b	CH_3OH	5.75 ± 0.31	$C_{56}H_{84}O_{21}S_2$	1157.4	20	115
Okadaic acid	CRM-OA-d	CH_3OH	8.4 ± 0.4	$C_{44}H_{68}O_{13}$	805.0	10	84
Dinophysistoxin-1	CRM-DTX1-b	CH_3OH	8.5 ± 0.7 [b]	$C_{45}H_{70}O_{13}$	819.0	10	85
	CRM-DTX1-c	CH_3OH	7.8 ± 0.5			10	78
Dinophysistoxin-2	CRM-DTX2-b	CH_3OH	3.8 ± 0.2	$C_{44}H_{68}O_{13}$	805.0	25	95
	CRM-DTX2-c	CH_3OH	3.83 ± 0.18			25	96
Pinnatoxin-G	CRM-PnTX-G	CH_3OH, 0.01% CH_3COOH	1.92 ± 0.09	$C_{42}H_{63}NO_7$	694.0	50	96
13-Desmethylspirolide C	CRM-SPX1-b	CH_3OH, 0.05% CF_3COOH	5.01 ± 0.24	$C_{42}H_{61}NO_7$	691.9	20	100
Gymnodimine-A	CRM-GYM-b	CH_3OH, 0.05% CF_3COOH	2.50 ± 0.13	$C_{32}H_{45}NO_4$	507.7	25	63

a - x_{CRM} = concentration of the CRM at 20°C, U_{CRM} = expanded CRM uncertainty
b - converted to µg mL^{-1}
c - injection volume lowered during method application to improve resolution with neighboring interference
d – see Table S2 for injector program
e - candidate CRM not released at the time of publication are shown with provisional property values and uncertainties, if available.
f – injection volume and associated ng on-column used only for within-class quantitation testing.

Table S2. Injection programs developed for large volume injections of samples. Diluent was drawn from vials filled with ultrapure water.

Injector Program		10 µL	15 µL	20 µL	25 µL	50 µL	72 µL
Action	Draw Sample (µL)	5	5	5	5	10	18
	Draw Diluent (µL)	20	20	20	18	10	9
	Draw Sample (µL)	5	5	5	5	10	18
	Draw Diluent (µL)	20	20	20	18	10	9
	Draw Sample (µL)		5	5	5	10	18
	Draw Diluent (µL)		20	20	18	10	9
	Draw Sample (µL)			5	5	10	18
	Draw Diluent (µL)			10	18	10	
	Draw Sample (µL)				5	10	
	Inject						

Table S3. LTQ XL electrospray ionization (ESI) tune parameters for ± polarities.

**Tune Setting (units)**	**ESI$^+$**	**ESI$^-$**
Source Voltage (kV):	3.50	2.70
Source Current (uA):	100.00	100.00
Capillary Voltage (V):	8.00	-36.00
Tube Lens (V):	85.00	-132.00
Skimmer Offset (V):	0.00	0.00
Multipole RF Amplifier	400.00	400.00
Multipole 00 Offset (V):	-1.75	5.00
Lens 0 Voltage (V):	-2.50	5.25
Multipole 0 Offset (V):	-5.00	5.75
Lens 1 Voltage (V):	-9.00	12.00
Gate Lens Offset (V):	-44.00	38.00
Multipole 1 Offset (V):	-11.00	9.00
Front Lens (V):	-5.25	5.75
Full Micro Scans:	1	1
Full Max Ion Time (ms):	200.00	200.00

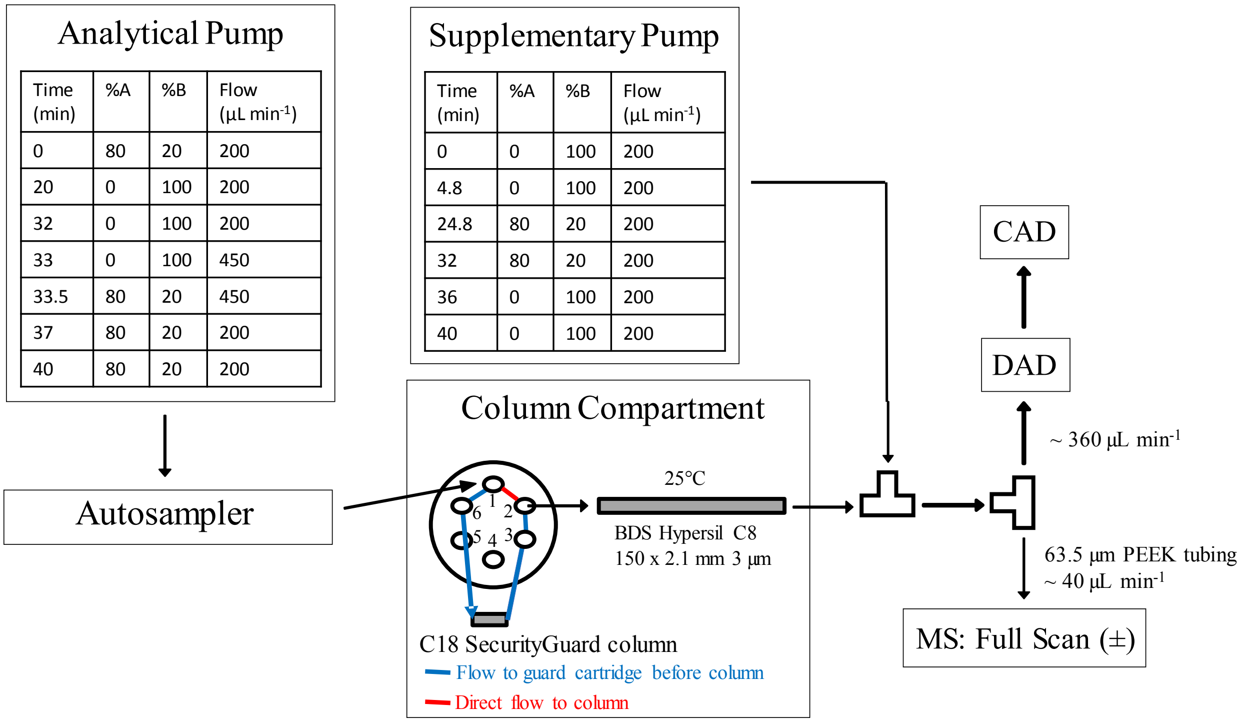

Figure S1. Schematic of LC–MS–DAD–CAD instrument configuration. Column compartment switching valve used to direct flow through two Phenomenex SecurityGuard C_{18} cartridges (4 × 3.0 mm ID) at 32 mins to clean it with 100% B following capture of mobile phase contaminants during re-equilibration of the previous run. PEEK tubing inner diameter was 127 µm unless otherwise indicated.

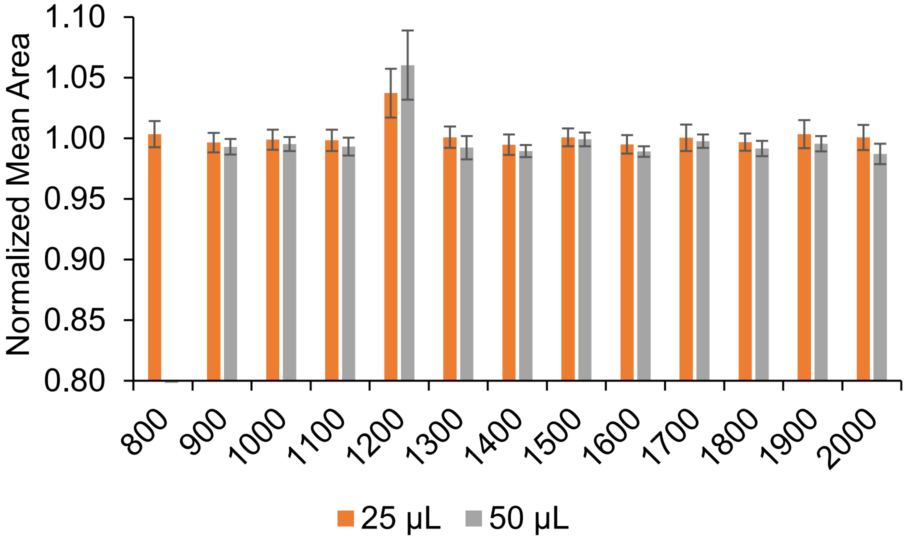

Figure S2. Normalized UV results from testing 5-fold and 10-fold dilutions of RM-RILC using 25 and 50 µL injection methods (Table S2). Results normalized to mean results from 5 µL standard injections of undiluted RM-RILC (n = 6 for all samples). Notes: 50 µL injection of RI 800 peak was excluded due to poor chromatographic peak shape; RI 1200 had an unresolved UV interference impacting its peak area.

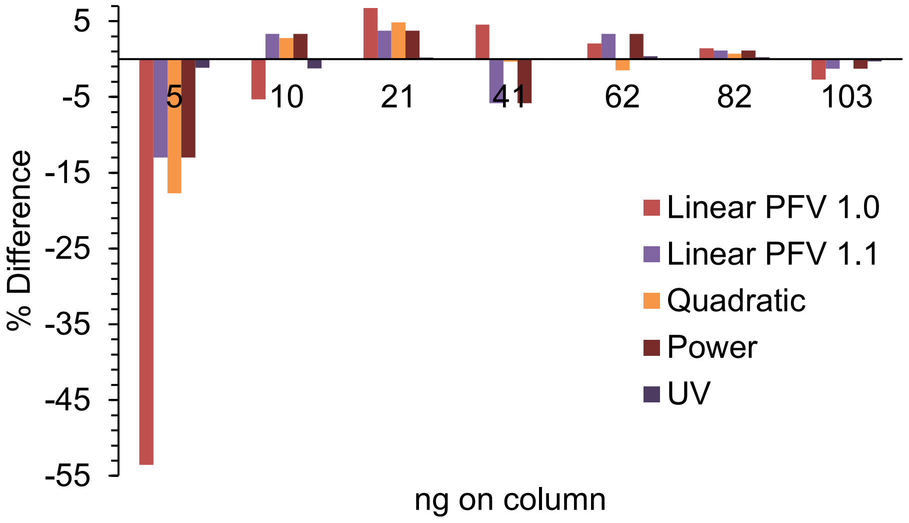

Figure S3. Difference (%) between the ng of MC-RR injected on-column and the calculated ng on-column using various regression models tested on the CAD data. Comparison with linear UV data from DAD detection also shown.

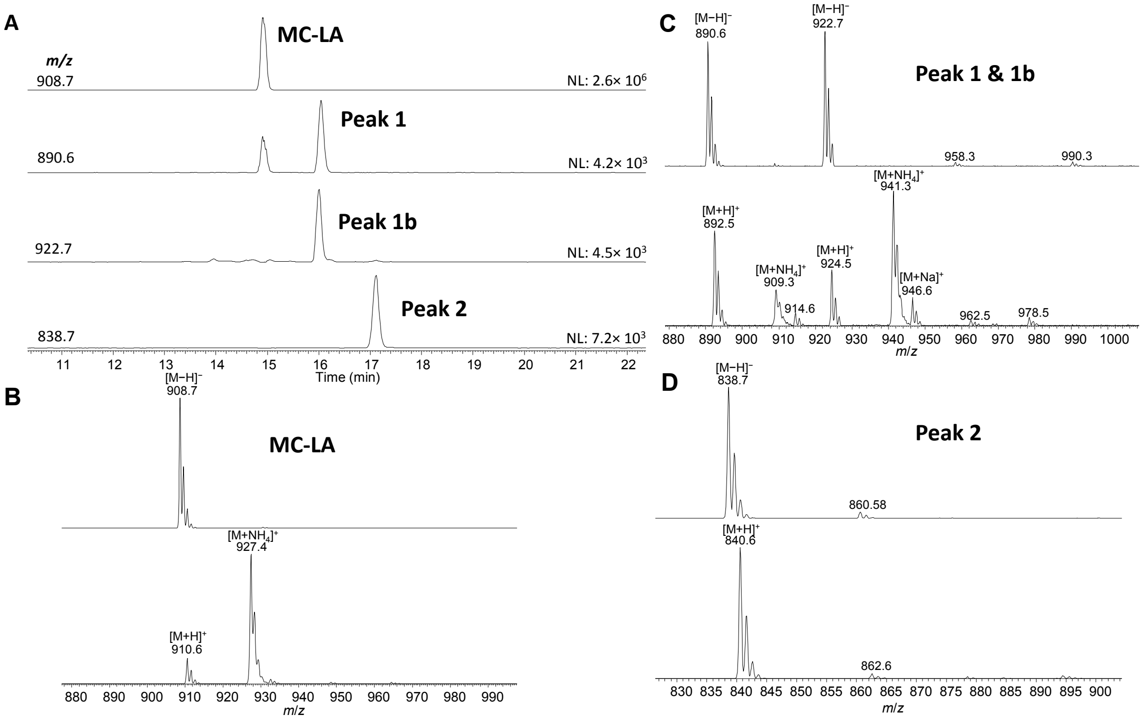

Figure S4. Analysis of candidate MC-LA CRM stock solution by full scan MS (LTQ XL). Negative mode chromatograms are shown (A) with normalized levels (NL) of intensity for each *m*/*z*, along with associated mass spectra from each peak (B, C, D) in both negative (top) and positive (bottom) polarities. Adduct distributions for peaks 1 and 1b are similar to that of MC-LA, while peak 2 does not show significant adduct formation.
